# Analysis of the Impact and Mechanism of Polyacrylate-Based Composite Paste on the Performance of Recycled Aggregate

**DOI:** 10.3390/ma17215242

**Published:** 2024-10-28

**Authors:** Huaisen Li, Chunhe Li, Hua Wei, Qingan Li, Hao Lu, Jinyu Ge

**Affiliations:** 1 Materials & Structural Engineering Department, Nanjing Hydraulic Research Institute, Nanjing 210029, China; lihuaisen_nhri@163.com (H.L.); w8880600@163.com (J.G.); 2 Civil and Environmental Engineering, The University of Miyazaki, 1-1 Gakuenkibanadainishi, Miyazaki 889-2192, Japan; lichunhe@cc.miyazaki-u.ac.jp; 3 Hanjiang Gushan Hydropower Development Co., Ltd., Wuhan 442631, China; liqingan_hghd@163.com

**Keywords:** recycled aggregate, composite slurry, coating modification, polyacrylate emulsion

## Abstract

This study developed three composite slurries for coating recycled aggregate by incorporating polyacrylate emulsion, fly ash, and gypsum into a cement-based mixture. The filling and pozzolanic effects of fly ash help to improve microcracks in the recycled aggregates. The polyacrylate emulsion forms a strong bonding layer between the cement matrix and the aggregates, enhancing the interfacial bond strength. Based on relevant studies, the following mix designs were developed: Slurry 1 consists of pure cement paste; Slurry 2 contains 15% fly ash and 3% gypsum added to the cement paste; Slurry 3 adds 22% polyacrylate emulsion to the slurry. The study first compared the effects of the three composite slurries on the crushing value and water absorption of recycled aggregates, and then analyzed their impact on the mechanical properties, permeability, and drying shrinkage of concrete. Finally, the mechanisms behind the enhancement were investigated using the Vickers Hardness Test (HV), Mercury Intrusion Porosimetry (MIP), and scanning electron microscopy–energy-dispersive spectroscopy (SEM-EDS). The results showed that the polyacrylate emulsion composite slurry had the most significant improvement effect. For recycled aggregate AL, the crushing value decreased from 28.8% to 22.5% and the saturated surface–dry water absorption decreased from 15.1% to 13.8% after cement slurry modification. After coating with the composite slurry, the crushing value further dropped to 18.2% and the water absorption to 9.5%. Two aspects of the performance of recycled aggregates are enhanced with the polymer composite slurry: first, fly ash provides nucleation sites for CH, reducing the tendency for directional CH alignment. Second, the long chains of PAE (polyacrylic ester) encapsulate cementitious particles, effectively filling surface defects on the recycled aggregates, improving the bonding strength at the new-to-old interface, and significantly enhancing the performance of both recycled aggregates and recycled concrete.

## 1. Introduction

With the rapid development of infrastructure and the renewal of old buildings, using recycled aggregates to partially or entirely replace natural aggregates in concrete has become a new research focus in engineering [1,2,3,4,5]. Compared to natural aggregates, recycled aggregates have undergone extensive weathering and erosion, leading to significantly reduced strength. Additionally, recycled aggregate often has more angular surfaces and numerous cracks, making it distinctly different in shape from natural aggregate. Furthermore, recycled aggregate typically has remnants of old cement mortar adhered to its surface. The interface between the old mortar and the aggregate, as well as the interface between the old and new mortar, creates weak interfacial transition zones (ITZ) [6,7,8]. This dual ITZ effect is a major factor contributing to the significant reduction in the performance of recycled concrete. Therefore, improving the interfacial properties of recycled aggregates is one of the key methods to enhance the performance of recycled concrete [9,10,11].

The current research is primarily focused on optimizing recycled aggregates by applying slurry coatings to their surfaces. Zhang et al. [12] used nano-silica and nano-calcium carbonate to prepare slurry coatings for recycled aggregates, finding that both types of nanoparticles effectively reduced the thickness of the interfacial transition zone (ITZ) in recycled concrete. Immersion treatments yielded better results. However, Chen Xuyong et al. discovered that an excessive amount of nano-silica in the mortar led to agglomeration, reducing the modification effect. Meng et al. [13] used a composite slurry made of mineral admixtures and nano-calcium carbonate to coat recycled aggregates, which significantly improved ITZ’s compactness. These studies demonstrate that composite cementitious materials containing admixtures can effectively reduce ITZ thickness and improve its surface properties. While these methods can modify recycled aggregates, they still face issues such as inadequate bonding strength and suboptimal modification effects. Studies have also explored the polymer modification of recycled concrete. Kou et al. [14] found that soaking recycled aggregates in a polyvinyl alcohol (PVA) solution significantly improved the shrinkage resistance and chloride ion penetration resistance of the resulting recycled concrete. Further studies mixed PVA solution with nano-silica to modify recycled aggregates, examining the effects of nanoparticles and polymers on recycled concrete’s properties. Geng et al. [15] reduced the water absorption of recycled aggregates by 36.5% through epoxy resin immersion, which also enhanced the mechanical properties of the recycled concrete. Santos et al. [16] investigated hydrophobic surface treatments by applying silane solution and paraffin wax to recycled aggregates. They found that both treatments reduced surface roughness, but paraffin wax was more effective in lowering aggregate porosity. Giri et al. [17] used an asphalt emulsion to coat recycled aggregates, producing a recycled asphalt mixture with comparable properties to natural crushed stone. Building on this, Ma et al. [18] incorporated waste cooking oil into the mixture, curing it at 160 °C for 1 h. They found that the oil residue reduced asphalt usage and improved the mixture’s fatigue resistance and low-temperature performance. Polymer immersion treatments can create a protective layer on the surface of recycled aggregates, but the bond between this polymer layer and the mortar remains weak.

Against this background, this study investigates the preparation of a polymer composite slurry using cement as the primary binder, with the addition of acrylic emulsion, fly ash, and gypsum. The cement paste enhances the mechanical and erosion resistance of aggregates by reducing interfacial cracks and promoting secondary hydration reactions. The filling and pozzolanic effects of fly ash help to reduce microcracks in the recycled aggregates and lower their water absorption rate. The acrylic emulsion forms a strong bonding layer between the cement matrix and the aggregates, partially mitigating the issue of weak interfacial bonding. As moisture evaporates, the acrylic emulsion in the polymer composite slurry accumulates on the surface of the recycled aggregates, forming a polymer film that makes the microstructure of the mortar denser and more uniform. Based on the relevant theoretical foundations, this study designed three types of polymer slurry to enhance the performance of recycled aggregates. The effects of the polymer composite slurry modifications on the performance of the resulting recycled aggregate concrete were tested to explore how each component contributes to strength improvements.

## 2. Raw Materials and Experimental Methods

### 2.1. Raw Materials

**Figure 1:**
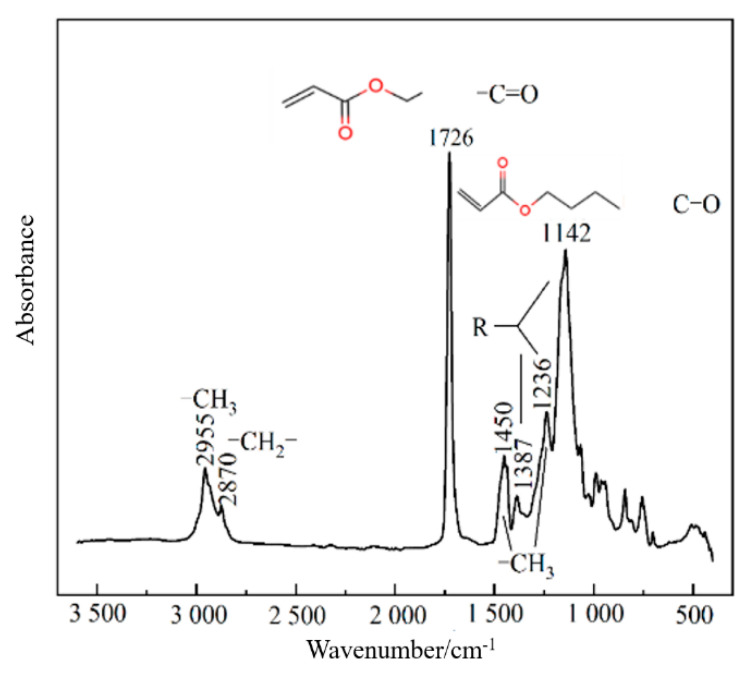
Infrared pattern of the modified polyacrylate polymer emulsion.

(1) Cement: The cement used in the experiment is Conch brand P·O 42.5 ordinary Portland cement. The basic physical properties of the cement are shown in Table 1.
(2) Mineral Admixtures: The fly ash used in this experiment is Grade I fly ash from Datang Nanjing Power Plant, with its basic performance indicators shown in Table 2. The gypsum used is produced by the Tangshan New Building Materials Factory in Jiangning District, Nanjing, and its basic properties are also listed in Table 3.
(3) Modified Acrylic Emulsion: The polymer used in this experiment is a modified acrylic emulsion produced by Nanjing Ruidi High-Tech Co., Ltd (Nanjing, China). The basic properties of the polymer emulsion are shown in Table 4; the infrared spectrum is shown in Figure 1. The carboxyl groups can form calcium salt complexes with Ca^2^⁺ in the cement hydration products, enhancing the mechanical properties of the slurry. The hydroxyl groups can physically adsorb onto cement particles through hydrogen bonding, improving the dispersion of the polymer in the cement matrix and increasing the bonding performance of the cement-based material.
(4) Aggregates: AL aggregates are produced by Changzhou Green Arrow Construction Waste Treatment Co., Ltd. (Changzhou, China) and are made by crushing low-grade concrete and red brick construction waste. AM aggregates are produced from crushed C30 concrete, and AH aggregates are produced from C40 concrete. The properties of the three recycled aggregates (AL, AM, and AH) used in this experiment are shown in Table 5, and their particle size distributions are presented in Table 6.
(5) Sand: River sand from Poyang Lake; water-reducing agent: polycarboxylate-based water-reducing agent; mixing water: tap water.

### 2.2. Experimental Methods

**Figure 2:**
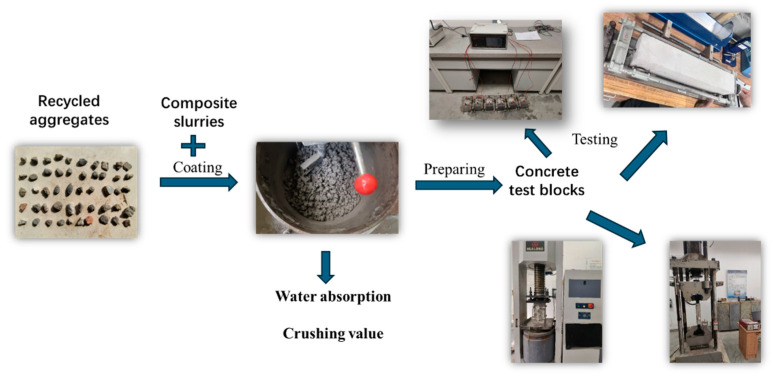
Recycled aggregate coating modification and modification performance process.

The experimental process of this research is shown in Figure 2. Firstly, according to the improvement effect that composite slurries have on the crushing value and water absorption of recycled aggregate, the modification effect of composite slurries on recycled aggregate is determined. Then, the compressive properties, tensile properties, elastic modulus, dry shrinkage resistance, and impermeability of the recycled concrete were measured to study the effect of composite slurries on the performance of recycled concrete.

#### 2.2.1. Composite Slurry Preparation

The mix proportions for the composite slurries, labeled as CE, MA, and PCP, are presented in Table 7: Slurry 1 is pure cement slurry, Slurry 2 is mixed with 15% fly ash and 3% gypsum in cement slurry, and Slurry 3 comprises the addition of 22% polyacrylate emulsion to Slurry 2. The method of coating the recycled aggregate with composite slurry was as follows: cement, fly ash, and gypsum were first weighed and dry-mixed in a mixer for 120 s. After this, the PAE emulsion and water were added, and the mixture was stirred for an additional 60 s to form the composite slurry. The recycled aggregate was then added to the mixer and stirred for 180 s to ensure it was coated with the composite slurry, resulting in slurry-coated recycled aggregate.

#### 2.2.2. Recycled Aggregate Concrete

Concrete mixes with different proportions were prepared using recycled aggregate coated with a composite slurry produced from CE and PCP. The experiment analyzed the performance of the slurry-coated recycled aggregate and its impact on concrete performance. Nine concrete mix designs were developed, varying according to the type of slurry coating and the strength of the recycled aggregate. Among these, RR served as the blank control group, CR was made using recycled concrete coated with CE slurry, and PR was made using recycled concrete coated with PCP slurry. The mix proportions are presented in Table 8.

Following the guidelines in the “Technical Specification for Testing and Inspection of Concrete in Hydraulic Engineering” (JTS/T 236-2019) [19], various tests were conducted on the recycled concrete, including tests of compressive strength, axial compressive strength, axial tensile strength, modulus of elasticity, impermeability, electrical flux, and drying shrinkage. Compressive strength was measured using a Shanghai Hualong WHY-3000 microcomputer-controlled pressure testing machine (Shanghai, China). Axial tensile strength was determined with a Donghua DHL-HLS concrete tensile universal testing machine (Suzhou, China). Impermeability values were measured using a Beijing Naierde NELD-S1005 mortar permeability tester (Beijing, China). Electrical resistivity was assessed with a Beijing Naierde NELD-PEU510 concrete electrical resistivity meter (Beijing, China). Drying shrinkage was measured using a Wafangdian Jianke HSY-540 concrete shrinkage-expansion tester (Wafangdian, China). All samples, except drying shrinkage samples, were cured under standard conditions for 28 days.

#### 2.2.3. Mechanistic Analysis

To clarify the mechanism by which composite slurry coatings improve the performance of recycled aggregates, low-grade aggregate AL was selected. Slurry mixes were prepared according to the proportions in Table 2 and applied to the surface of recycled aggregates to create test samples. The samples were cured for 28 days, freeze-dried, and subjected to further characterization and testing. The specific experimental procedures are as follows:

(1) Micro-morphology and Microhardness: The micro-morphology of the interfacial transition zone (ITZ) in recycled aggregates was observed using an HVS-1000 digital microhardness tester (Shanghai, China), and the microhardness of the interface region was measured. The tests were conducted with a constant load of 50 g, applied for 10 s. To prevent the concentration of stress due to the densely packed sampling points, which could affect the accuracy of the results, a staggered arrangement method was used to select the test points.
(2) Porosity and Pore Structure: The porosity and pore structure of the samples were characterized using Mercury Intrusion Porosimetry (MIP). The MIP analysis was conducted using a Micromeritics AutoPore IV 9510 (Shanghai, China), which operates in a pressure range of 0.0037 to 241.1 MPa and measures pore diameters from 340 to 0.005 μm.
(3) Microstructure: The microstructure and elemental distribution in specific regions of the samples were analyzed using Scanning Electron Microscopy with Energy Dispersive Spectroscopy (SEM-EDS). The testing was performed using a JEOL JSM-5900(Richland, WA, USA). Prior to testing, the sample surfaces were gold-coated to enhance their conductivity.

## 3. Test Results and Analysis

### 3.1. Modified Recycled Aggregate Performance Analysis

**Figure 3:**
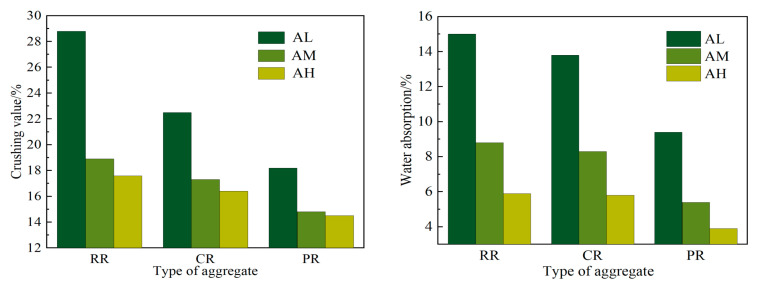
Water absorption and crushing value of recycled coarse aggregate.

The recycled aggregates obtained from AL, C30 (AM), and C40 (AH) were treated using three methods: no treatment (RR), coating with 0.5 mm pure cement slurry (CR), and coating with 0.5 mm composite slurry (PR). After curing for 28 days, their crushing values and water absorption rates were tested. The results are shown in Figure 3.

It can be observed that, for the AL aggregate, the crushing value decreased from 28.8% to 22.5% and the saturated surface–dry water absorption rate decreased from 15.1% to 13.8% after modification with pure cement slurry. The reductions were 21.9% and 8.0%, respectively. For AM and AH aggregates, reductions were also observed in crushing value and saturated surface–dry water absorption rate. The crushing value for AM decreased from 18.9% to 17.3%, and for AH it decreased from 17.6% to 16.4%, representing reductions of 8.5% and 6.8%, respectively. The saturated surface–dry water absorption rate for AM decreased from 8.9% to 8.3%, and for AH it decreased from 5.9% to 5.8%, with reductions of 5.7% and 1.6%, respectively.

After coating with composite slurry, the crushing value of AL aggregate decreased from 28.8% to 18.2%, and the saturated surface–dry water absorption rate decreased from 15.0% to 9.5%, with reductions of 36.8% and 34.0%, respectively. For AM and AH aggregates, there were also significant reductions in both crushing value and saturated surface–dry water absorption rate. The crushing value for AM decreased from 18.9% to 14.8%, and for AH it decreased from 17.6% to 14.5%, representing reductions of 21.7% and 17.6%, respectively. The saturated surface–dry water absorption rate for AM decreased from 8.8% to 6.1%, and for AH it decreased from 6.0% to 4.2%, with reductions of 30.7% and 28.8%, respectively. These data indicate that coatings with low-water–cement-ratio slurry can reduce the crushing value and saturated surface–dry water absorption rate of recycled aggregate. However, coatings with composite slurry showed a more significant improvement in both properties compared to pure cement slurry, with a more pronounced reduction in the saturated surface–dry water absorption rate.

### 3.2. Performance Analysis of Recycled Aggregate Concrete

#### 3.2.1. Mechanical Property

**Figure 4:**
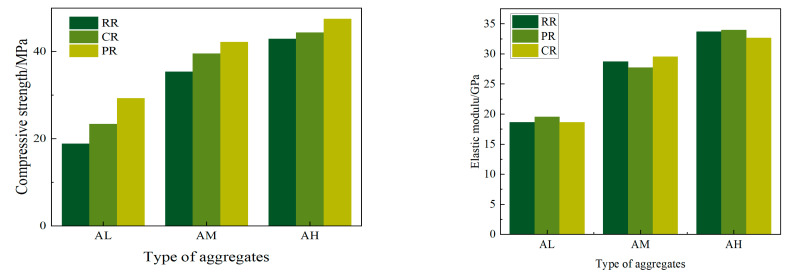
Compressive strength and elastic modulus of recycled concrete.

Compressive strength tests were conducted on recycled concrete with different aggregates, and the results are shown in Figure 4. Regarding compressive strength, the test results indicate that as the strength of the recycled aggregate increases, the improvement in concrete strength due to the slurry coating gradually decreases. For CR-AM and PR-AM, the strength increased by 4.1% and 13.6% compared to RR-AM, respectively. For CR-AH and PR-AH, the strength increased by 5.2% and 9.9% compared to RR-AH, respectively. Since the AL aggregate has the highest crushing value and saturated surface–dry water absorption rate among the three groups, at 28.8% and 15.0% respectively, it can be inferred that the strength of the AL aggregate is lower than that of AM and AH.

The test results for compressive strength indicate that as the strength of the recycled aggregate increases, the improvement in concrete strength due to the slurry coating gradually diminishes. Specifically, CR-AM and PR-AM showed strength increases of 4.1% and 13.6% compared to RR-AM, respectively, while CR-AH and PR-AH increased the strength by 5.2% and 9.9% compared to RR-AH, respectively. Since the crushing value and saturated surface–dry water absorption rate of the AL aggregate are the highest among the three groups, at 28.8% and 15.0%, respectively, it can be concluded that the strength of the AL aggregate is lower than that of AM and AH.

**Figure 5:**
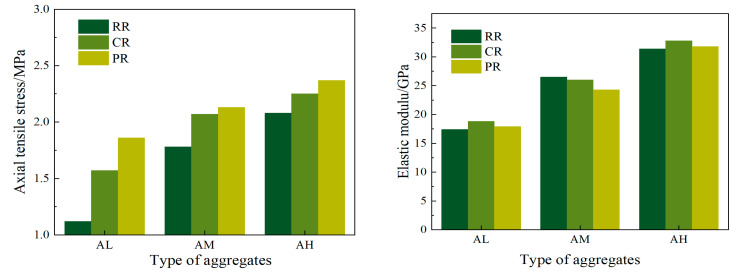
Axial tensile and elastic modulus of recycled concrete.

Tensile strength tests were conducted on recycled concrete with different aggregates, and the results are shown in Figure 5. For tensile strength, it is evident that the axial tensile strength of RR-AM and RR-AH increased by 58.9% and 85.7%, respectively, compared to RR-AL. Similarly, CR-AM and CR-AH increased the axial tensile strength by 31.9% and 43.3%, respectively, compared to CR-AL, while PR-AM and PR-AH increased it by 14.5% and 27.4%, respectively, compared to PR-AL. This indicates that using a composite slurry coating can reduce the differences in the tensile strength of recycled concrete due to the variations in recycled aggregate strength. Additionally, compared to RR-AM, CR-AM, and PR-AM increased the axial tensile strength by 16.3% and 19.7%, respectively, while CR-AH and PR-AH increased it by 8.2% and 13.9%, respectively, compared to RR-AH. This suggests that as the strength grade of recycled aggregate increases, the effectiveness of a slurry coating in improving the tensile strength of recycled concrete gradually decreases.

Regarding the elastic modulus, it can be observed that when the mix proportion of recycled concrete is fixed, the variation in the elastic modulus is minimal. This indicates that the elastic modulus is not significantly influenced by the slurry coating process but is primarily dependent on the inherent strength and elastic modulus of the recycled aggregate.

The results of the above experiments can be summarized as follows: the slurry coating process has a more pronounced impact on the mechanical properties of recycled concrete made with lower-grade recycled aggregates. However, as the strength grade of the recycled aggregate increases, the influence of the slurry coating process on the mechanical properties of the recycled concrete diminishes. Additionally, for the same recycled aggregate, the use of a polymer-enhanced composite slurry more significantly improves the performance of the recycled aggregate and leads to greater enhancements in the mechanical properties of the recycled concrete.

#### 3.2.2. Permeability Test

**Figure 6:**
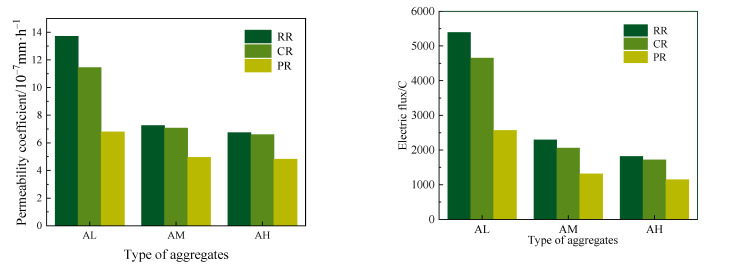
Permeability coefficient and electrical flux of recycled concrete with different aggregates.

Permeability and electrical conductivity tests were conducted on recycled concrete made with aggregates of varying strength grades, with the results shown in Figure 6.

For the permeability coefficient, it was observed that the values for RR-AL, CR-AL, and PR-AL were 13.71 × 10^−7^ mm/h, 11.45 × 10^−7^ mm/h, and 6.79 × 10^−7^ mm/h, respectively. Compared to RR, the CR and PR values decreased by 13.1% and 48.4%, respectively. For RR-AM, CR-AM, and PR-AM, the permeability coefficients were 7.25 × 10^−7^ mm/h, 6.74 × 10^−7^ mm/h, and 4.95 × 10^−7^ mm/h, respectively, with CR and PR showing reductions of 7.0% and 31.2% compared to RR. Similarly, for RR-AH, CR-AH, and PR-AH, the permeability coefficients were 6.91 × 10^−7^ mm/h, 6.59 × 10^−7^ mm/h, and 4.81 × 10^−7^ mm/h, respectively, where the CR and PR values were reduced by 4.6% and 30.4% compared to RR.

For electrical charge passed (coulombs), the values for RR-AL, CR-AL, and PR-AL were 5394C, 4654C, and 2568C, respectively. Compared to RR, the CR and PR values were reduced by 13.7% and 52.4%, respectively. For RR-AM, CR-AM, and PR-AM, the electrical charge passed was 2298C, 2059C, and 1117C, respectively, with CR and PR showing reductions of 10.4% and 42.7% compared to RR. Similarly, for RR-AH, CR-AH, and PR-AH, the electrical charge was 1816C, 1720C, and 1147C, respectively, with CR and PR reducing the charge by 5.3% and 36.9% compared to RR.

The experimental results indicate that both the cement paste and polymer composite slurries significantly improved the impermeability of recycled concrete. The enhancement is especially pronounced with polymer composite-coated recycled aggregates, which have a more substantial effect on the permeability of recycled concrete made from aggregates of the same strength grade. However, as the strength grade of the recycled aggregates increases, the effectiveness of the paste coating in improving impermeability gradually diminishes.

#### 3.2.3. Drying Shrinkage

**Figure 7:**
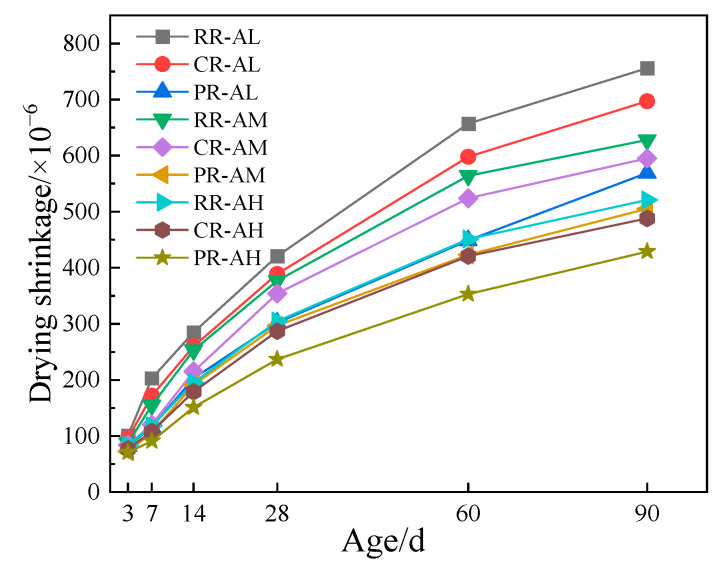
Drying shrinkage of recycled concrete with different aggregates.

Drying shrinkage tests were carried out on recycled concrete with different aggregate strength grades, and the results are shown in Figure 7.

The shrinkage values varied significantly across different mixed ratios. Compared to RR-AL, PR-AL reduced shrinkage rates by 19.0%, 42.9%, 28.8%, 28.3%, 31.7%, and 24.6% at 3, 7, 14, 28, 60, and 90 days, respectively. Similarly, PR-AM showed reductions of 17.2%, 31.6%, 24.5%, 21.2%, 25.0%, and 19.6% compared to RR-AM. For PR-AH, the reductions were 15.5%, 22.8%, 22.5%, 22.3%, 21.7%, and 17.6% compared to RR-AH over the same time periods.

The shrinkage test results show that applying the slurry coating reduces the shrinkage rate of recycled concrete, indicating that the composite slurry coating effectively decreases shrinkage across different aggregate strength grades. The influence of the slurry coating on shrinkage performance diminishes as the aggregate strength increases. This is mainly because, at the same water–cement ratio, shrinkage performance is primarily related to the porosity and the elastic modulus of the aggregate. The elastic modulus of recycled concrete made with AM and AH aggregates is significantly higher than that made with AL aggregates. Therefore, as the aggregate strength increases, the higher elastic modulus of the recycled aggregate can effectively counteract the cement paste’s tendency to shrink due to water loss, thereby reducing the drying shrinkage of the recycled concrete.

### 3.3. Discussion

The surface of recycled aggregates is rough and porous, with remnants of old mortar, which leads to high water absorption and poor interfacial bonding. Coating the recycled aggregates with cement paste forms a film that seals the pores, reducing water absorption and improving the crushing value of the aggregates. The mechanism behind using fly ash and gypsum as mineral additives to enhance the performance of recycled aggregates lies in their filling ability. Fine particles of fly ash can fill the pores and microcracks in the aggregates, thereby reducing porosity and water absorption, increasing density, and improving crushing strength. Gypsum reacts with C_3_A in the cement to form ettringite, which helps fill microcracks and improves the microstructure of the recycled aggregates [20]. Compared to the CE modification, the water absorption of AL recycled aggregates modified with MA was reduced by 1.4%, and the crushing value decreased by 1.2%. Furthermore, compared to MA, the PCP-modified AL recycled aggregates showed a further reduction in water absorption by 0.6% and a decrease in crushing value by 2.4%. It is evident that fly ash primarily reduces water absorption in modified recycled aggregates, while polymers improve strength by enhancing the crushing value [21]. This provides valuable guidance for optimizing composite slurry formulations for specific construction scenarios.

The mechanism that can improve the performance of slurry-modified recycled aggregate concrete lies in an enhancement in the bonding strength at the new-to-old interface and reinforcement of the weak zones. When coated with cement slurry, the Ca (OH)_2_ reacts with the unhydrated silicates in the old cement paste, leading to secondary hydration and the formation of new hydration products, such as C-S-H gel. These newly formed hydration products not only fill the pores on the surface of the recycled aggregates but also increase the bond strength between the aggregates and the cement matrix, enhancing the concrete’s strength and durability. The pozzolanic reaction of fly ash consumes the Ca (OH)_2_ generated during hydration, while gypsum regulates the hydration rate of C_3_A, and the resulting ettringite fills the voids. This synergy significantly improves the interfacial transition zone, pore structure, and durability of recycled aggregate concrete [22]. Compared to AL aggregates modified with CE, the compressive strength of recycled concrete increased by 4.1% with MA and 13.6% with PCP. The addition of polymers further enhanced the compressive strength. The tensile strength increased by 58.9% with MA and 85.7% with PCP, though the addition of polymer did not significantly further improve the tensile strength. The permeability coefficient decreased by 7.0% with MA and 31.2% with PCP, with the addition of polymer noticeably further reducing permeability. The electrical charge decreased by 13.7% with MA and 52.4% with PCP, with the polymer addition significantly further lowering the electrical charge. The reason for this is that the acrylic emulsion forms a strong bonding layer between the cement matrix and aggregates, improving interfacial bonding and reducing the potential for crack propagation [23]. When recycled concrete aggregates are used, this enhanced bonding ability helps to address the weak interfacial properties of the recycled aggregates. By reducing the porosity in the mortar, the acrylic emulsion improves the mortar’s impermeability and electrical resistivity. The film-forming effect of the acrylic emulsion creates a more uniform and denser microstructure in the mortar, effectively improving compressive, tensile, and flexural strength while reducing internal cracking.

The analysis revealed that mineral additives primarily enhance the crushing value of recycled aggregates through the filling ability of fly ash and the hydration-promoting effect of gypsum. The polyacrylate component reduces water absorption, mainly through its film-forming and filling properties. The improvements in the mechanical properties of recycled concrete are mainly attributed to the adhesive and film-forming effects of the polyacrylate emulsion, which enhances compressive and tensile strength. Durability improvements are mainly due to secondary hydration and the filling of microcracks, which increase impermeability and reduce the electrical charge. The combined use of polyacrylate emulsion presents a clear advantage over other polymers in enhancing bond strength, as it forms an effective bonding layer with cement-based materials, addressing the shortcomings in interfacial bonding.

## 4. Mechanism Analysis

To clarify the mechanism behind the performance enhancement of recycled aggregates coated with composite slurry, low-strength AL aggregates were selected. The slurries were prepared using the proportions shown in Table 8. A mix of different proportions of recycled concrete (kg/m^3^) was applied to the surface of the recycled aggregates to create the test samples. These samples were cured for 28 days, then freeze-dried, and subsequently characterized and tested. The results and analysis are as follows.

### 4.1. Micro-Morphology and Microhardness

**Figure 8:**
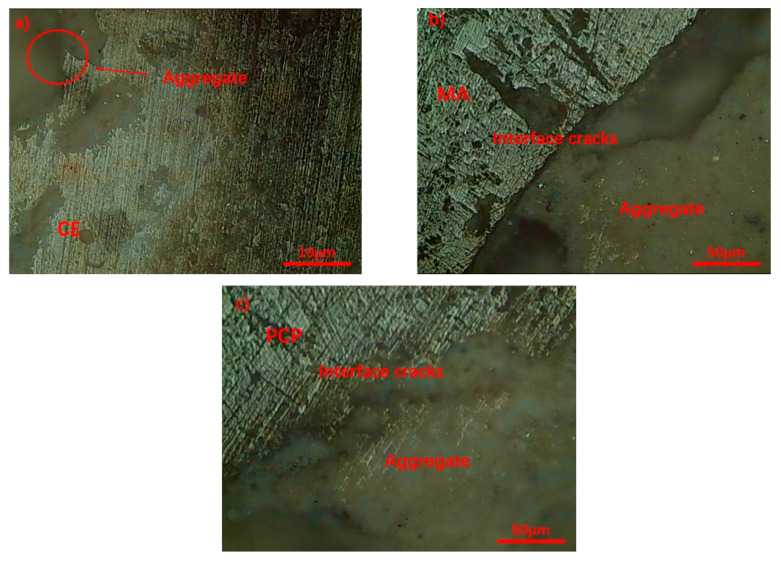
Micro-morphology of the interface transition zone: (**a**) ×40 CE; (**b**) ×200 MA; (**c**) ×200 PCP.

The microstructure of the interfacial transition zone (ITZ) was observed, and the results are shown in Figure 8. Significant cracks appear at the CE and MA interfaces. However, after the incorporation of the PAE emulsion, the bond between the recycled aggregate and the paste becomes much tighter, with no clear interface or visible cracks in the ITZ. This indicates that PAE emulsion effectively improves the adhesion between the paste and the recycled aggregate. Furthermore, the structure near the PAE-modified ITZ shows fewer pores and reduced crack defects, demonstrating that PAE can effectively reduce porosity and enhance the performance of the ITZ.

**Figure 9:**
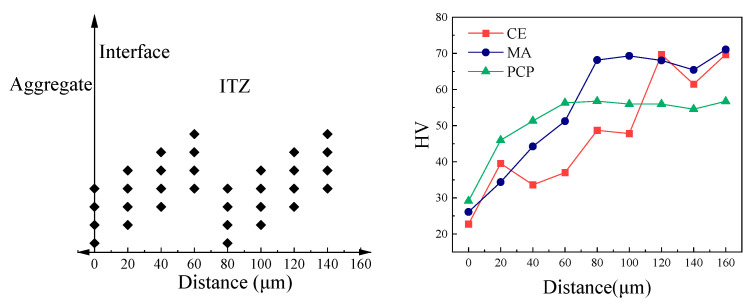
Microhardness sampling points and results.

Figure 9 shows that the microhardness of the PCP sample at the interface is HV 29.10, slightly higher than that of MA (HV 26.08) and CE (HV 22.73), with increases of 11.6% and 28.0%, respectively. As the distance from the interface increases, the microhardness of all samples also increases. For PCP, the microhardness stabilizes at 60 μm, while for MA, microhardness stabilizes at 80 μm, and CE exhibits significant fluctuations, reaching a maximum at 120 μm. The addition of fly ash and gypsum reduces the thickness of the interfacial transition zone (ITZ), and PAE further reduces it. Notably, at 20 μm from the interface, the average microhardness reaches HV 45.96, close to the average microhardness of the PCP paste (HV 56.05). This suggests that PAE combined with fly ash and gypsum not only reduces the ITZ thickness but also improves its performance. Additionally, the microhardness of the composite paste is lower than that of CE, likely due to the lower stiffness of the PAE film, which reduces the overall stiffness of the composite paste. Up to 60 μm, the microhardness of PCP remains higher than that of CE and MA. Since the ITZ is generally the weakest zone in recycled concrete, significantly affecting its mechanical and durability properties, it can be inferred that the composite paste improves recycled concrete’s performance by reducing ITZ porosity and increasing its microhardness.

### 4.2. Porosity and Pore Structure

**Figure 10:**
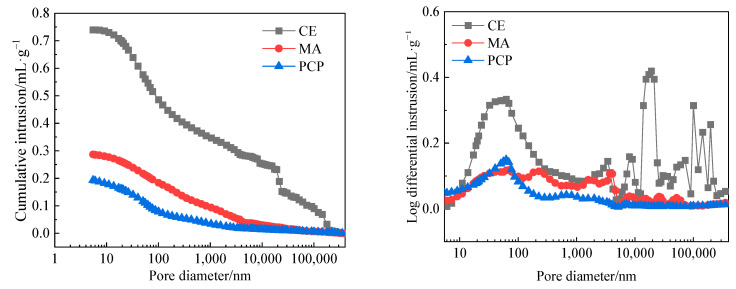
Microscopic pore distribution test results of recycled aggregate after coating modification.

The MIP analysis results for the samples are shown in Figure 10. The cumulative mercury intrusion volumes for CE, MA, and PCP are 0.74 mL/g, 0.29 mL/g, and 0.19 mL/g, respectively. This indicates that the inclusion of fly ash and gypsum significantly reduces the micro-porosity of modified recycled aggregates, while the addition of PAE further decreases the micro-porosity. For MA, the reduced cumulative porosity, in conjunction with the SEM and XRD results, reveals that on the one hand, fly ash particles delay the early hydration reaction of cement, enhancing its fluidity and effectively filling the recycled aggregate interface, thus reducing the interface’s porosity. On the other hand, fly ash particles react in the cement environment, forming hydration products such as C-S-H, C-S-A, and C-A-H, and dissolving zeolite-type crystals. Gypsum promotes the formation of AFt from C-A-H in the cement, consuming CH and further reducing the surface porosity of the aggregate. For PCP, the reduced cumulative porosity is mainly due to the PAE emulsion, which, in an alkaline environment, has a disrupted surface charge. PAE long chains are released and re-crosslinked to form a network polymer film within the cement paste, encapsulating the cement clinker particles and hydration products. As hydration progresses, water in the polymer film is continuously absorbed by the cement clinker particles, leading to an increase in particle volume and the formation of a denser network polymer film, which further reduces the porosity of the aggregate interface.

In comparison, PCP has a smaller pore size distribution. This indicates that using pure cement paste leads to larger pores in the interface region of recycled aggregates. The addition of fly ash and gypsum effectively improves the pore refinement on the aggregate surface, while PAE significantly enhances the refinement of capillary pores [24]. Combining the XRD and SEM analyses, it is evident that fly ash and gypsum physically fill the surface cracks and consume CH to form dense gel, whereas PAE creates a dense polymer film that encapsulates hydration products, further increasing their density. Among the samples, the third peak, in terms of peak intensity, is ranked as PCP, MA, CE, indicating that PCP contains the highest proportion of gel pores, followed by MA and CE, which contain the least. This confirms that PCP has the highest number of gel-like substances, followed by MA and CE, which have the least. Comparing MA and CE, it can be observed that the capillary pores on the aggregate surface are partially filled, likely due to the fine, dense microspheres in fly ash. The addition of PAE further fills the large pores on the aggregate surface and enhances capillary pore refinement [25]. PAE long chains effectively penetrate capillary pores on the aggregate surface, crosslink, and form a dense polymer film, which effectively fills and tightens capillary pores [26]. These conclusions regarding the pore structure align with the observed effects of different composite slurry coatings on the performance of recycled aggregate concrete.

### 4.3. Analysis of Microstructure

To further understand the slurry coating process and the effect of each component on the interfacial transition zone, the samples were analyzed using SEM, and the microscopic morphology and elemental distribution of the “recycled aggregate–slurry interface” were analyzed as follows.

**Figure 11:**
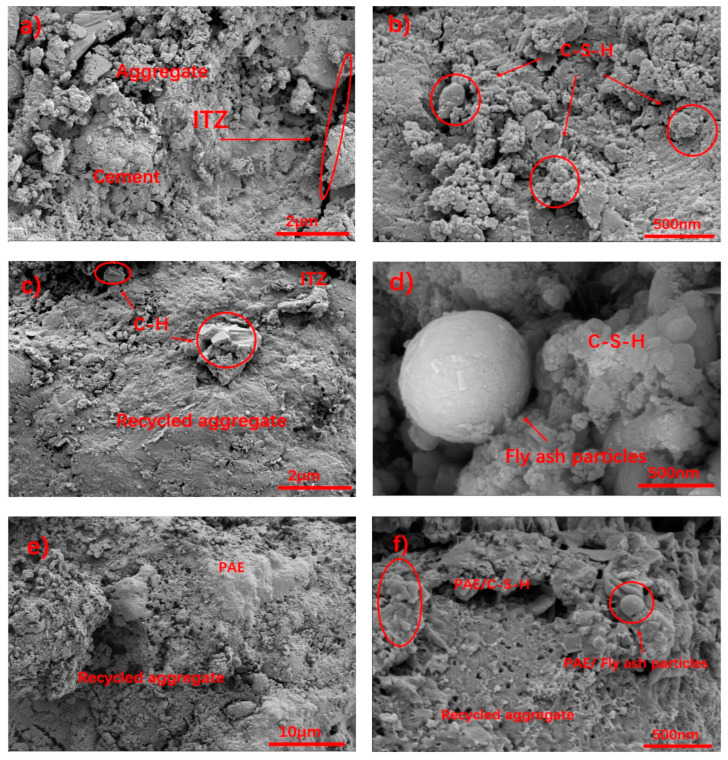
Microscopic morphology of the interface transition zone. (**a**) ×5000 CE; (**b**) ×20000 CE; (**c**) ×5000 MA; (**d**) ×20000 MA; (**e**) ×1000 PCP (**f**) ×20000 PCP.

The microscopic morphology of the CE samples is shown in Figure 11a,b. It is evident that there are significant cracks between the aggregate surface and the interface transition zone, indicating poor adhesion between the CE slurry and the transition zone. Figure 11b shows numerous fine cracks in the CE interface transition zone, with cracks extending along the direction of the aggregate surface, further indicating the prevalence of gaps in the transition zone. This reveals that the interface transition zone is primarily composed of multiple layers of plate-like hydration products mixed with amorphous gel. The CH crystals are hexagonal prisms aligned parallel to the aggregate surface, which grow into plate-like crystals perpendicular to the hexagonal prism faces, i.e., {001} plane growth. During the cement hydration process, different ions dissolve and migrate at different rates [27], resulting in the early accumulation of a large amount of Ca^2+^ and OH- near the aggregate. These ions start their nucleation and growth on the aggregate surface, forming thin layers of CH early in the hydration process. As hydration progresses, a large amount of SiO_3_^2−^ migrates to the aggregate surface. Since SiO_3_^2−^ promotes the growth of CH along the {001} planes [28], Ca^2+^ and OH- precipitate on the CH layer, leading to the gradual growth of plate-like hexagonal prisms.

The microscopic morphology of the MA samples is shown in Figure 11c,d. Figure 11c reveals that fly ash effectively fills the surface of the recycled aggregate and the interface transition zone, penetrating the internal defects of the aggregate and providing a physical filling. This Figure also shows that the transition zone is mainly composed of amorphous hydration gel, AFt, fly ash particles, and layered CH. The amorphous hydration gel is primarily C-S-H, indicating that the pozzolanic effect of fly ash converts some CH into C-S-H, thereby enhancing the mechanical properties of the transition zone and reducing the risk of severe cleavage due to CH’s crystal orientation. As shown in Figure 11d, with the addition of fly ash and gypsum, the CH crystal morphology in the MA samples significantly changes compared to that in the CE samples. Specifically, the crystals are further refined, and their arrangement becomes more disordered. This refinement may result from the compression of CH crystals due to the growth of C-S-H gel, leading to deformation or fragmentation of the crystals. Additionally, during the hydration process, gypsum dissolves in the slurry to form SO_4_^2−^ ions, which can inhibit the growth of CH along the {001} planes [29]. As a result, CH crystals are less likely to develop into highly oriented structures.

An observation of the PCP interface transition zone, as shown in Figure 11e,f, shows that fly ash microspheres suffered from dissolution damage, and were surrounded by the C-S-H gel encapsulated by polymer films. Initially, fly ash particles physically filled the surface cracks in the recycled aggregate. As hydration proceeded, the fly ash underwent pozzolanic reactions, gradually dissolving to form C-S-H [13]. Additionally, during hydration, the long chains of PAE hydrolyzed to generate carboxyl groups, which then formed a complex with Ca^2+^ ions to form a dense structure. Consequently, the PAE long chains tightly adhered to the C-S-H gel surface. As hydration continues, the PAE chains cross-linked and aggregated to form a polymer film that enveloped the C-S-H gel. The C-S-H gel continued to grow and absorb free water from the PAE film, which dehydrated to form a dense membrane, ultimately creating a compact structure that further filled the aggregate surface defects and reduced the porosity of the interface transition zone [30]. Figure 11f shows that the PAE film covered the surface of the fly ash and effectively filled the defects in the recycled aggregate, confirming the earlier conclusions about the synergistic effect of fly ash and PAE. The images reveal that, with the addition of the PAE emulsion, the density of the PCP interface transition zone improves further. There are almost no visible cracks between the composite slurry and the recycled aggregate, and the boundary between the aggregate and the transition zone is less distinct [31]. This indicates that the PAE emulsion further reduces defects between the interface transition zone and the recycled aggregate, thereby enhancing the bond between the recycled aggregate and the slurry [32].

## 5. Conclusions

Based on the analysis of the effects of various composite slurry formulations on the performance of recycled aggregates and the prepared recycled concrete, as well as the underlying microscopic mechanisms, the following conclusions can be drawn:

(1) The application of paste coating methods effectively reduces the crushing value and water absorption rate of recycled coarse aggregates. Compared to the CE modification, the water absorption of AL recycled aggregates modified with MA was reduced by 1.4%, and the crushing value decreased by 1.2%. Furthermore, compared to MA, the PCP-modified AL recycled aggregates showed a further reduction in water absorption of 0.6% and a decrease in crushing value of 2.4%. It is evident that fly ash primarily reduces water absorption in modified recycled aggregates, while polymers improve aggregate strength by enhancing the crushing value. This provides valuable guidance for optimizing composite slurry formulations for specific construction scenarios.

(2) Coating recycled aggregates with different paste materials significantly enhances the mechanical properties, durability, and shrinkage resistance of the resulting recycled concrete. Among these, the use of polymer composite slurry coatings achieves the most pronounced improvements, including a 54.5% increase in compressive strength, a 66.1% increase in axial tensile strength, a 46.2% reduction in permeability coefficient, a 52.4% reduction in electrical flux, and a 24.7% reduction in 90-day drying shrinkage. Comparing recycled aggregates of varying strength levels, the improvement in performance is more significant for recycled concrete made with lower-strength-grade aggregates, with the effect diminishing as the aggregate strength increases.

(3) The performance enhancement of recycled aggregates using polymer composite slurry is attributed to the synergistic effects of its components. Fly ash reduces the tendency of CH (calcium hydroxide) to orient by providing nucleation sites and chemically reacting to consume some CH in the interfacial transition zone (ITZ). Gypsum dissolves early in the hydration process and reacts with CH to form AFt (ettringite), thereby reducing the proportion of CH in the ITZ. PAE (polyacrylate ester) emulsion, when emulsified in an alkaline environment, releases long-chain PAE molecules that wrap around cement particles, effectively filling the surface defects in the recycled aggregates. This process also forms a dense network polymer membrane through complexation, enhancing the microscopic bonding effects between the paste and the aggregates. Ultimately, this results in a significant improvement in the related properties of the recycled aggregates and the recycled concrete.

## Figures and Tables

**Table 1:** P·O 42.5 basic physical properties of cement.

Density (g/cm^3^)	Specific Surface Area (m^2^/kg)	Standard Consistency Water Consumption (%)	Initial Setting Time (min)	Final Setting Time (min)
3.03	355	27.5	195	305

**Table 2:** Basic properties of fly ash.

Density (g/cm^3^)	Specific Surface Area (m^2^/kg)	Fineness (45 μm Square Hole Sieve Residue) (%)	Water Demand Ratio (%)
2.24	542.17	10.4	91

**Table 3:** Gypsum’s basic properties.

Liquidity (mm)	Initial Setting Tiame (min)	Final Setting Time (min)	Compressive Strength (2 h) (MPa)	Flexural Strength (2 h) (MPa)
178	10	20	11.6	4.3

**Table 4:** Properties of modified acrylic polymer emulsion.

Solid Content (%)	Density (g·cm^−3^)	Viscosity (Pa·s)	pH	Grain Size (μm)	MFT (°C)
45	0.94	3.60	7.8	0.1~0.2	7~10

**Table 5:** Different properties of recycled aggregates.

Samples	Saturated Surface Dry Apparent Density (kg/m^3^)	Water Absorption (%)	Crushing Value (%)
AL	2120	14.96	28.8
AM	2411	5.01	18.9
AH	2444	4.93	17.6

**Table 6:** Recycled coarse aggregate particle gradation.

Samples	20~16 mm	16~10 mm	10~5 mm	<5 mm
AL	6.66	81.48	11.56	0.3
AM	7.69	75.16	16.95	0.2
AH	6.91	79.22	14.66	0.2

**Table 7:** Proportions of the three composite slurries.

Samples	Cement (g)	Fly Ash (g)	Gypsum (g)	PAE (g)	Water (g)
CE	300	0	0	0	75
MA	246	45	9	0	75
PCP	246	45	9	66	35.4

**Table 8:** Mix proportions of recycled concrete (kg/m^3^).

Samples	Cement	Recycled Aggregates	Sand	Water	CE	PCP	Water-Reducing Agent
RR-AL	437.5	1113.0	495	175	0	0	7
CR-AL	437.5	1113.0	495	175	163.4	0	7
PR-AL	437.5	1113.0	495	175	0	163.3	7
RR-AM	437.5	1046.9	495	175	0	0	7
CR-AM	437.5	1046.9	495	175	144.1	0	7
PR-AM	437.5	1046.9	495	175	0	144	7
RR-AH	437.5	1034.5	495	175	0	0	7
CR-AH	437.5	1034.5	495	175	144.3	0	7
PR-AH	437.5	1034.5	495	175	0	144.8	7

## Data Availability

The original contributions presented in the study are included in the article, further inquiries can be directed to the corresponding authors.
